# E‐cigarettes, vaping and performativity in the context of tobacco denormalisation

**DOI:** 10.1111/1467-9566.12741

**Published:** 2018-04-17

**Authors:** Mark Lucherini, Catriona Rooke, Amanda Amos

**Affiliations:** ^1^ Usher Institute of Population Health Sciences and Informatics University of Edinburgh Edinburgh UK

**Keywords:** e‐cigarettes, smoking, performativity, renormalisation, young adults, qualitative research

## Abstract

E‐cigarettes are devices through which a nicotine solution is ‘vapourised’ and inhaled by the user. Unlike cigarettes, the process involves no tobacco combustion. However, the inhalation and exhalation of vapour is reminiscent of smoking and there is debate about the possible harms and benefits of e‐cigarette use, including the ‘renormalisation’ of smoking. Despite these debates, there has been little exploration into the embodied and semiotic similarities between smoking and vaping. This paper views the practices of vaping and smoking through the lens of performativity that is, the accumulation of meaning associated with the habits over time and space. Through in‐depth interviews, we explore how young adults from primarily disadvantaged areas in Scotland, understand the similarity in practices between smoking and vaping. Participants talked about financial barriers to using different types of e‐cigarettes, and how their use reflected their views on smoking cessation. They also discussed the embodied similarities between smoking and vaping, with divergent opinions on whether this continuance of habit was beneficial or not, revealing still developing and ambiguous norms around performativity. The norms of vaping were also frequently discussed, with participants’ experiences and views reflecting the contested position of vaping in an environment where cigarette smoking is denormalised.

## Introduction

Denormalisation is a term used to encompass the various strategies of making smoking a less normal social activity and is commonplace in many western countries (Chapman and Freeman 2008). At a policy level these strategies involve tax increases on cigarettes, smokefree legislation, graphic packet warnings, plain packaging and mass media campaigns and they work in tandem with ‘social denormalisation’ (Hammond *et al*. 2006), aimed at changing social attitudes to smoking. In Scotland, the effects of these strategies can be seen in the falling rates of adult smoking, from 28 per cent in 2003 to 21 per cent in 2016 (McLean *et al*. 2017). However, the social outcome of denormalisation has situated smoking and smokers as marginal (Bell *et al*. 2010a) and, in turn has led to a self‐stigmatisation (Evans‐Polce *et al*. 2015, Ritchie *et al*. 2010a), and the development of smokers as a socially sanctioned ‘other’ in public spaces (Dennis 2016). Denormalisation has also highlighted distinct socio‐economic disparities in which smoking is clustered in the most disadvantaged areas, where the activity is more normalised (Barnett *et al*. 2017, Robinson and Holdsworth 2013) and those already marginalised bear the brunt of stigma (Graham 2012, Thompson *et al*. 2007). In Scotland, for example, 35 per cent of adults in the most deprived areas are smokers, compared to 11 per cent in the least deprived (McLean *et al*. 2017). These moral and ethical consequences of denormalisation have long stoked debate among those working in tobacco control research. Some have questioned ‘absolutist’ views on stigma as either good or bad (Bayer 2008), and contend that stigma is contextual and can be deployed to positive effect in public health (Bayer and Fairchild 2015). However, others suggest that stigma, even in softer forms, continues to marginalise rather than empower those who are particularly susceptible to stigmatisation, such as disadvantaged populations (Williamson *et al*. 2014) and so increases health inequalities (Bell *et al*. 2010b). Some have also argued that public health objectives, to reduce and ultimately eradicate tobacco smoking, have been prioritised at the expense of more nuanced understandings of why people continue to smoke in the face of clearly disseminated evidence of harm (Bell 2011; Dennis 2016). There have therefore been calls to focus tobacco control measures away from direct denormalisation of smoking, by tackling the impoverished social circumstances that are so connected with smoking in the first place (Graham *et al*. 2006). This paper considers perspectives on social denormalisation, in light of the proliferation of electronic cigarettes (e‐cigarettes), in countries where smoking denormalisation is present, such as the UK, and what this might mean for those working in public health policy and practice.

## E‐cigarettes

E‐cigarettes are handheld devices that come in a variety of shapes and sizes. The devices heat a nicotine solution until it is vapourised and inhaled by the user. Early models introduced in the 2010s, commonly termed ‘cigalikes’, closely resembled cigarettes and were often pre‐filled and disposable. Recently, more sophisticated models, termed ‘vapourisers’ or ‘mods’, have developed, which can be customised to suit individual preferences. These later models can be filled with selected nicotine solutions, or e‐liquids, which come in a variety of strengths and range from ‘tobacco’ to candy flavours. Specialist ‘vape’ shops are now a common feature of the retail landscape.

E‐cigarettes have been quickly subsumed into public health debates, reflecting the way in which Dennis (2016) describes tobacco research as being subsumed by public health agendas at the expense of more sociological perspectives. This is not surprising given the potential of the devices for smoking cessation and harm reduction. For instance, in England, Beard *et al's* (2016) findings from a large time‐series dataset show a correlation between increased use of e‐cigarettes and increased quit success but, whether or not this is due to e‐cigarettes remains unclear. E‐cigarettes have ‘disrupted’ (Stimson *et al*. 2014) existing views on tobacco control strategies in many western countries due to their ambiguous possibilities, including the possibility of renormalising tobacco smoking, and providing a ‘gateway’ to smoking (Britton *et al*. 2014, Chapman 2014, Hall *et al*. 2015, Sweanor 2015). Furthermore, the impact of e‐cigarettes on existing smoking inequalities is unclear (Hartwell *et al*. 2017, Kalousova 2015). In a rejection of the idea that smoking may be renormalised through vaping, Voigt (2015) draws upon a common sense argument in which vaping and smoking are viewed as distinctly different activities. Similarly, Britton *et al*. (2014) argue that the visibility of vaping normalises vaping, not smoking. The lack of any substantive evidence that vaping impacts on smoking denormalisation is often referenced in defence of this view (Bauld *et al*. 2015).

Further supporting the idea that e‐cigarettes are a distinct product and vaping a distinct activity from smoking and so not a route for smoking renormalisation, Measham *et al*. (2016), used participatory methods with a large sample of UK teenagers, and found that e‐cigarette use was initiated for the desirable flavours and the novelty of the products. Drawing on small groups discussions in the US, Roditis and Halpern‐Felsher (2015), proposed that lack of knowledge about e‐cigarettes led teenagers to have more positive opinions about the devices. Likewise, the young adult (aged 18–30) participants in Coleman *et al*.'s (2016) US focus groups, were mostly approving of e‐cigarettes as replacements for tobacco cigarettes, noting a shedding of stigma when they were used in public places. However, other research has suggested that smokers’ understandings of e‐cigarettes are not clear‐cut. Rooke *et al*. (2016) found, in focus group discussions, that UK adult smokers’ understandings and uses of e‐cigarettes can be dependent on their smoking history and status, such that a ‘spectrum’ of views exist. In ethnographic research in a working‐class area in Northern England, Thirlway (2016), found that the meanings attached to e‐cigarettes were tied up with issues of morality and gender, with a ‘functional’ use – for smoking cessation – being approved of, and ‘recreational’ use – for the purposes of creating ‘smoke clouds’ – being disparaged. Building on this emerging body of research, we explore how meaning is attached to e‐cigarettes and vaping through performative similarities and differences, especially among young adults from disadvantaged backgrounds, where smoking is relatively more prevalent but still subject to disapproval and denormalisation.

## Performativity of smoking and vaping

Butler (2006) considers performativity to be constituted by a series of socially constructed practices that have become relatively stable in a certain context. Butler discussed gender, but her work has been developed in various directions and performativity can be extended to the ‘performances’ of smoking that have developed over time in ‘smokefree’ countries such as the UK. For instance Dirksmeier and Helbrecht (2010) discuss the importance of interpersonal encounters in shaping the performativity of urban spaces. For smoking this is exemplified by Poland (2000) who explains that the performance of the ‘considerate smoker’; who deliberately withdraws to marginal spaces to smoke, decides not to smoke around others and may ask permission from non‐smokers before lighting a cigarette, has become an internalised, expected performance for many smokers and non‐smokers. It is in the repetition of these practices, which come to legitimise them as the ‘natural’ way of smoking (Butler 2006). The emergence of vaping is however creating a new type of interpersonal encounter for which the performativity is uncertain. Gregson and Rose (2000), have focused on space itself as a primary agent in shaping performativity. Indeed, the ‘smokefree era’ (Dennis 2016) in many countries with advanced tobacco control policies, shapes this performativity, creating public spaces where ‘clean’ atmospheres are expected (Tan 2013) and through which spaces of smoking are ‘shrinking’ (Collins and Procter 2011). Tan (2013) is concerned that this will create increasingly sterile urban environments where the vibrancy of sensorial diversity is lacking but wonders if denormalisation and exclusion of smokers can or should ever be challenged.

Performativity is not always adhered to unquestioningly but is a self‐managed practice in the face of social context. Goffman (1990) considers social interaction as performances in which people must manage their ‘presentation of self’ to others, especially through limiting the negative social impacts of potentially stigmatising characteristics. Of course, demure smoking is not the only performativity, especially for young people. Scheffels (2009) noted, through interviews with 18–23 year old Norwegian smokers, that although denormalisation had served to stigmatise smokers in public space, smoking remained a way to perform a transition into adulthood. Likewise, Rooke *et al*. (2013) noted, through a range of qualitative methods, that the smokefree legislation in the UK which saw smoking become an outdoor practice led to some young adults using the process of going outside to smoke to form a sociable identity with other smokers. Therefore, denormalisation does not always necessarily lead to stigmatising outcomes.

Following the influential work of (Graham 1993) who, through qualitative and quantitative investigation, highlighted that smoking was being used as a coping mechanism among low‐income women in the UK, others have explored the meaning of smoking among disadvantaged communities and shown that the social circumstances of life further direct the performativities of smoking. Robinson and Holdsworth (2003) conducted biographical interviews and ethnography in working‐class communities in Liverpool, and found that cigarettes were often important cultural and social commodities made meaningful by being shared with neighbours, especially at times of distress. Farrimond and Joffe (2006) using innovative qualitative interview techniques with UK adults, revealed how socio‐economic status (SES) can impact on performativity. They found that smokers from lower SES backgrounds were more likely to internalise markers of smoking stigma, and less likely to assert a more positive smoking self‐identity, than more affluent smokers. This is evident in other qualitative research with adults which found disadvantaged smokers in the UK considering themselves ‘lepers’ (Ritchie *et al*. 2010a), while middle to higher income smokers in New Zealand were able to deploy self‐management strategies to reduce the stigmatising impact of smoking, for example, by rationalising the extent of their addiction (Thompson *et al*. 2009). This highlights a difference by SES in the resources smokers can mobilise in their ‘presentation of self’ (Goffman 1990). While disadvantaged smokers are conscious of smoking stigma, more affluent smokers are not only able to avoid this double stigma but utilise a more rational explanation for continued smoking. Social inequalities are additionally linked with the spatial performativity of smoking, as it is increasingly restricted to areas of disadvantage. Thompson *et al*. (2007), for example, described ‘smoking islands’: urban areas in New Zealand where statistics show high rates of smoking and socio‐economic disadvantage. Qualitative interviews revealed local people undergoing a compounding stigma of social disadvantage and continued smoking in the face of anti‐smoking public health campaigns.

E‐cigarettes enter this context rather ambiguously. Bell and Keane (2012: 245) argue that e‐cigarettes may be viewed negatively because of their mimicry of smoking: the production of an exhaled vapour ‘is an unmistakable signifier of smoking and therefore invokes both the memory of public smoking culture and its possible resurgence’. A sense of disapproval, therefore, may transfer from cigarettes to e‐cigarettes due to their semiotic similarities, with less importance placed on their technical differences. Ahmed's (2004: 91–92) discussion of performativity, is helpful to understand that the performativity of smoking may also ‘stick’ to vaping; an example of Ahmed's conceptualisation of the performative as:

Futural: it generates effects in the constitution or materialisation of that which is ‘not yet’. But on the other hand, performativity depends upon the sedimentation of the past; it reiterates what has already been said, and its power and authority depend upon how it recalls that which has already been brought into existence.

Those who see performative similarities between smoking and vaping draw on this ‘sedimentation of the past’, pointing to the established performativity of smoking. Others deny the importance of performativity ‘sticking’ by stressing that objective differences, once fully understood, will be privileged over performativity (Britton *et al*. 2014, Voigt 2015). Performativity can be drawn on to understand responses to previous tobacco control interventions, for example, in observational research exploring how smokers transitioned their smoking practices to outdoor spaces in the wake of the indoor smoking ban in the UK (Ritchie *et al*. 2010b). Likewise, through, approaching smokers in urban environments without prior arrangement to conduct ‘ethnographic interviews’, Bell *et al*. (2015) considered the embodied strategies smokers in the UK, US, Canada and Australia, used to minimise the disruption of graphic pack warnings to smoking habits, such as skilfully handling (‘handiness’) packets to avoid seeing images. Vaping can also be less spatially restricting that smoking, as through focus groups with male US teenagers, Peters *et al*. (2013) found that compared to cigarettes, e‐cigarettes were used in smokefree contexts easily, and ‘expeditiously’. In this study, we focus on the embodied and spatial aspects of performativity to explore how young adults from disadvantaged areas in Scotland are making sense of vaping.

## Methods

### Study design

Twenty‐two small group interviews and 11 individual interviews were conducted with 16–24 year olds from primarily disadvantaged communities in central Scotland between August 2015 and April 2016. Small friendship group interviews have been used successfully in previous research with young people about substance use (Highet 2005). They enable dialogue to occur between participants, for example challenging and/or supporting each other's accounts, rather than participants only responding to the interviewer (Highet 2003). Participants were recruited through community organisations and educational institutions. Recruiting from disadvantaged areas can be challenging as participants are often socially and economically marginalised, therefore staff (usually older adults) at organisations were initially approached. These gatekeepers often organised the friendship groups as they knew participants well and, due to certain marginalised social circumstances of some participants, gatekeepers, advised on the suitability of either individual or group interviews. In order to reach participants in education and employment, a request was placed on the skills exchange website Gumtree. Respondents were asked to invite one or two friends to join the interview. However, some preferred one‐on‐one interviews with the researcher.

Before deciding to take part, participants were given an information sheet detailing what the interview would involve and written consent was obtained. Ethical approval was obtained through the University of Edinburgh, Centre for Population Health Sciences Ethical Review Group. Interviews were conducted by one of the authors at community venues and participants’ homes. Participants were given a £15 voucher for contributing to the research. Interviews lasted on average 50 minutes and were recorded. The interviews were semi‐structured and the topic guide covered questions on smoking/vaping history, and where, when and how participants had encountered e‐cigarettes.

**Figure 1:**
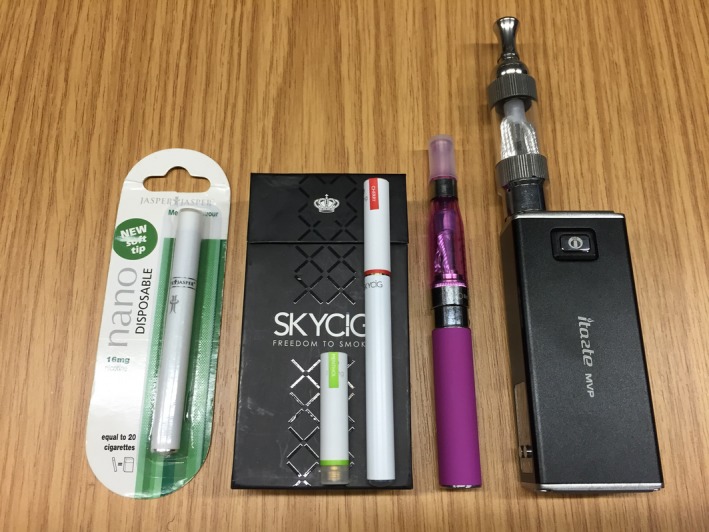
E‐cigarette props used in interviews. From left to right: disposable cigalike, reusable cigalike with interchangeable flavours (packet contents on exterior), vapouriser, box mod.

The interviewer brought examples of the three main e‐cigarette types as props for participants to examine: disposable and reusable ‘cigalikes’, a pen‐shaped ‘vapouriser’ and a box‐shaped ‘mod’ device (Figure 1). This was especially useful as participants found the embodied and spatial performative uses of e‐cigarettes easier to convey using these props. The props also created complementary ethnographic data. For instance, at times participants expressed disgust at the e‐cigarettes by contorting their faces or physically recoiling from the table on which they were displayed. The interviewer also used visual examples of e‐cigarettes being used in various settings, and e‐cigarette advertisements. Using a method developed by Rooke *et al*. (2016), statement cards about popular e‐cigarette opinions, paraphrased from media coverage, were used to promote conversation if it became strained or difficult.

### Participants

There were 72 participants in total. Thirty‐nine participants were female and the average age was 19.6 years. There were 44 current smokers, 13 ex‐smokers and nine ever‐smokers. Due to the friendship group method the researcher often had little control over who participants or gatekeepers invited to take part. Therefore, there were also five never‐smokers and one participant of undetermined smoking status. Most participants (60) had tried vaping at least once. Fourteen were current vapers, five were ex‐vapers and ten had never tried vaping. The vaping status of two participants was undetermined. Undetermined smoking/vaping status was due to some participants contributing little to the discussions or providing vague and/or contradictory answers. On the advice of gatekeepers about the social marginalisation of some participants, and for good ethical practice, the interviewer did not press for responses when participants were not forthcoming.

Participants were recruited from a range of educational/employment backgrounds. Nearly half (31) were not in employment, education or training (NEET), 18 were in education and 18 were in part‐time or full‐time employment. The remaining five were in volunteer or peer‐training programmes. The dominance of NEET participants reflected the social profile of Scottish smokers, with those from more deprived areas more likely to be smokers (McLean *et al*. 2017). Socioeconomic data was not formally recorded but many participants reflected on the normality of smoking in their communities, families, peer networks and age group. Non‐smokers also talked about ‘battles’ to refrain from smoking given its ubiquity in their social worlds. The responses of smokers and non‐smokers are considered together given their similar social contexts and experiences with smoking.

### Analysis

The interview data was transcribed verbatim and imported into Nvivo V.10 (QSR International, Brisbane) for analysis. A thematic analysis was initially carried out by the authors to identify recurring themes (Braun and Clarke 2006). This analysis was guided both etically from the research questions of the project but also emically to allow the researcher to identify other themes relevant to the research aims (Crang and Cook 2007). Themes around the habits of smoking and vaping were identified and subsequently a lens of performativity, drawing from the insights of Ahmed (2004), was applied to conduct a more nuanced theoretical coding (Braun and Clark 2006), on which this paper reports.

## Findings

Contrasting participant understandings of how smoking and vaping relate are presented, starting with the performative differences of varying types of e‐cigarette, followed by the significance of embodied similarities of smoking and vaping. A discussion of how the participants felt about the spatial performativities of vaping follows. The findings are presented and discussed around quotes and excerpts from participants. Some excerpts represent individual perspectives, while others represent the conversational nature of group interviews, in order to convey the context of the discussion, but also demonstrate that e‐cigarettes and the norms of vaping were only beginning to be considered among the participants. Participants have been given pseudonyms, with their smoking status, vaping status and age in brackets.

## Performativities of different types of e‐cigarette

It is important to underline that e‐cigarettes are not a homogenous product and research has tended to gloss over the attributes of different types, especially how they relate to meaning making around the devices and their use (Thirlway 2015). Different types of e‐cigarette were used in different ways by the participants and vaping performativity was not uniform. Most participants had limited experience with the advanced e‐cigarette types and often preferred cigalikes. Following examination of the box mod (Figure 1) some of these less experienced vapers felt that the performativity of vaping should follow smoking:

He [friend] has these big huge things [box mods] and I'm like ‘what even is that, like, it doesn't fit discreetly in your pocket?’ (Melissa, ex‐smoker, never‐vaped, 22).

People hold them [box mods] in their hand at bus stops, and I'm like can you not just stick to the wee skinny ones? … That's not even like holding a fag. (Justine, smoker, ever‐vaped, 16).

As Farrimond and Joffe (2006) find, smokers from low SES backgrounds are more likely to be aware of the negative identity markers that smoking brings in the smokefree environments of the UK, suggesting that the norms of the ‘considerate smoker’ (Poland 2000) may be linked with circumstances of disadvantage. Melissa and Justine are unsettled by ostentatious vaping in public spaces that comes with the box mods, as this goes against ingrained norms of ‘considerate’ smoking and so, cigalikes allowed a presentation of the self which aligned with existing smoking norms, in which discretion prevails.

Despite commenting on the favourable design of cigalikes, many of the less experienced vapers still believed them to be the least effective for smoking cessation: ‘I think it's cool; I think it's well done but I don't think they work compared to the pens and the box ones’ (Helen, smoker, ever‐vaped, 22). The price of the more advanced types was also a common point of discussion among participants who viewed the most effective devices for smoking cessation as, for many, unaffordable:

If you want a decent one then they're like 40 to 60 quid [pound sterling] out your pocket. And then sometimes you can't afford that. You're just scraping together your coppers [coins] to get a ten deck [pack of cigarettes] (Allison, smoker, ever‐vaped, 19).

Perhaps the higher price of advanced models and the ready availability of cigalikes ‐ ‘the wee things that look like cigarettes you can go in and buy them out of pound shops’ (Nina, smoker, vaper, 24) – meant that smokers on low incomes were more exposed to cigalikes and low‐end vapourisers. Therefore smoking inequalities, such as Kalousova (2015) suggests, may be reproduced through pricing and availability of different types of e‐cigarette.

Participants felt that vapourisers and box mods enrolled a different set of performativities. For example, Peter (smoker, ex‐vaper, 16) felt it was acceptable to use his mod device on the train due to the different way the device was held: demonstrated by his gripping of the box mod prop in his full hand, rather than held between fingers, like he would with the other e‐cigarette models. For Nina, the vapouriser enabled a concealable behaviour and so allowed her to avoid some of the stigma she experienced when she was seen smoking with her children: ‘It's smaller, easier to hold, easier to hide out the way … if you don't want people to see you obviously smoking’. Sarah (smoker, vaper, 24) spoke of the satisfaction of creating large smoke clouds: ‘the amount of smoke you get from it is so much more satisfying than a … cigarette, you get way more smoke and it's not disgusting … it looks really cool’. As Dennis (2016) discusses, the exhalation of second‐hand smoke is almost entirely negative for non‐smokers but retains a pleasurable release for smokers who can ‘travel’ with the outbound smoke. However, for Sarah, there is a satisfying release of vapour that carries with it less stigma than a similar smoke release. While Nina and Sarah had different perspectives on being seen vaping they both demonstrated how the different performativities of vaping provided more spatial options than smoking, whether through ‘expeditious’ (Peters *et al*. 2013) use or through a shedding of disgust (Coleman *et al*. 2016). Sarah touches on vaping as being ‘cool’, much like some young people can use smoking for reasons of youthful identity formation (Scheffels 2009). However, the sense of vaping as cool was not particularly strong among the sample with most noting that it was other people they knew who used e‐cigarettes for this reason, especially those younger than themselves.

Vapourisers and box mods were often considered the most effective types for quitting smoking. Graham (smoker, ex‐vaper, 19) mentioned an acquaintance and disparaged his recreational use of box mods:

I know somebody that's got one and they just use it for blowing clouds … what's the point in that, you just look like a tool. So I bought that one [vapouriser] because like actually the reason why I bought it was for the purpose … to actually stop [smoking].

This reflects Thirlway's (2016) suggestion that the ways of using e‐cigarettes can differ by social class: among Thirlway's, mostly working‐class, sample, e‐cigarettes were used and talked about functionally – for smoking cessation – rather than recreationally. The performance of maturity evident in Graham's response was also evident in Thirlway's study, especially among men, for whom e‐cigarettes could be a ‘badge of moral intent’ (p.110) indicating control and action over their attempts to quit smoking.

## Embodied performativity

The participants generally knew little about the technical aspects of e‐cigarettes when it came to relative harm and they often asked the interviewer for clarification:

Peter Is that absolutely worse for you than smoking cigarettes … do you know?

Interviewer Um, nobody knows

Peter Nobody knows?

Interviewer There's not been … enough research on it to actually say. I think the sort of general conclusion is that they're probably not as bad for you.

Not wanting to influence their opinions or claim to have medical expertise, the interviewer gave non‐committal replies to these questions. Like in Roditis and Halpern‐Felsher's (2015) research, exchanges like this highlighted the lack of knowledge about e‐cigarettes among young people but also their curiosity to find out more. Their understanding of relative harm, therefore, was influenced by the performativities of smoking and vaping. This echoes Bell's (2011: 50) proposal that public health measures to curb second‐hand smoking are partly based on ‘the subjectively experienced abjectness of cigarette smoke far more than the “objectively” demonstrable harms to health it causes’. Like cigarette smoke, the vapour of e‐cigarettes served to pollute the air for many of the participants and they often dismissed the chemical and material differences between smoke and vapour:

Gloria (ex‐smoker, ever‐vaped, 19) I don't like it … because I don't like smoke, so I don't like that [vapour].

Leslie (ever‐smoker, ever‐vaped, 16) Like if I was sitting having my dinner and somebody was smoking one of them [an e‐cigarette] …

Gloria It would put you off.

Leslie I know.

Interviewer Even though it's a kind of different smell and it's not…?

Gloria It's still smoke.

Leslie It's still smoke.

The performative similarities extended to the processes of inhalation and exhalation as some participants felt that vaping was little different to smoking due to the process of inhalation. Justine and Viccy had remarked that others using e‐cigarettes were not cautious enough of the possible dangers and suggested that vaping was akin to smoking. Seeking clarification, the interviewer asked:

So, you think it's just not different from smoking a cigarette?

Viccy (undetermined smoking status, never‐vaped, 16) No.

Justine (smoker, ever‐vaped, 16) Well, it is.

Viccy Well, yes, it is, I suppose, obviously, yes.

Justine Because the chemicals, but it's like you're still doing…you're still breathing in some sort of thing.

Justine and Viccy reasoned that there is an obvious difference between smoking and vaping but the process of inhaling something, most likely harmful, into your body was a key similarity for both. Dennis (2016) discusses how the inside of bodies have come to be enrolled as smokefree by visceral anti‐smoking advertising that shows the journey of smoke into bodies such that we are now more conscious of what we inhale into our body interiors. Many of the participants, like Justine and Viccy, demonstrated how this internal smokefree realm has developed into a vapefree space, into which nothing ‘chemical’ or unknown should be breathed.

Exhalation was also considered by the participants:

Melissa (ex‐smoker, never vaped, 22) My pal smoked this one [points to the box mod] and it produces, like, a lot of smoke, like, loads …

Rachel (smoker, never vaped, 24) Aye that's what it was like.

Melissa … looks like a cloud!

Rachel I know, that's why I didn't like when his [referring to her infant son] dad smoked it around him ‘cause it was, like, a lot of smoke going in his face.

Bell (2011) notes that the disapproval aimed at second‐hand smoke is often rooted in the potential harms to children and other vulnerable, innocent groups. The participants in this study were similarly concerned about second‐hand vapour, and demonstrate how the ‘explication of the air’ (Dennis 2016) has become a cornerstone of tobacco denormalisation, as they are distrustful of any ‘smoky’ substance that might transgress their own bodily boundaries, and those of their children. Vaping therefore often involved the same contaminating potential as smoking. Dennis points out that it is not necessarily the harm of second hand smoke that causes people to recoil from smoking bodies, but the relative disgust at ingesting a substance that has already circulated through the body of a smoker. Interestingly, e‐cigarettes could also reverse the meaning of this circulation into a positive sensory experience, and create a pleasurable sensory landscape, which as Tan (2013) describes can improve experience of urban environments. Fred demonstrated this potential, when he talked about encountering second‐hand vapour:

Fred (smoker, vaper, 24) If you're on the other side [of the pavement] and somebody's walking round [vaping], you're more inclined to walk behind them to get that puff, and you're like [makes pleasurable inhaling/smoking noises]. It's like going into the petrol station when you're a wee [young] boy, and you're like [makes noises again].

E‐cigarettes were often understood as cessation aids and some felt that the similarity of vaping to smoking would be beneficial compared to most licensed nicotine replacement therapies:

Interviewer Do you think you would try and quit smoking at some point in the future?

Clarissa (smoker, ever vaped, 18) Aye, I'd probably give it a bash [try], now, with one of them. But before, I wouldn't have done it, with just like the chewing gum, or anything like that. But now that they've actually got, like, wee things [e‐cigarettes] that are like smoking, then aye, I probably would try it now.

Conversely, some participants felt that the similarity was not beneficial:

I didn't find it was helping me like stop smoking, I was still having a fag, and then when I finished the fag … just started vaping on that [e‐cigarette]. It's more, for me it's like the hand‐mouth action thing. That's why I find it didn't really help me that much (Donald, smoker, vaper, 18).

Not only was the inhaling and exhaling of vapour similar to smoke but the hand‐to‐mouth habit also endured. These aspects are the same, regardless of the type of e‐cigarette being used and therefore, for many participants ‘It's the same thing. It's still smoke. It stinks.’ (Laura, smoker, ever‐vaped, 17). The majority of participants did comment on the similar performativity, but, like Clarissa and Donald, they differed on whether this was good or bad in terms of smoking cessation.

## Developing spatial norms for vaping

A key way that participants discussed performativity was in conversation about whether vaping is acceptable indoors, particularly in smokefree spaces. Justine was talking about an image of young men vaping in a pub:

Justine I think that's bad, smoking in the pub.

Interviewer But these are vaporisers rather than cigarettes.

Justine Aye, but I don't think that's nice … it's not nice to look at, you know. You wouldn't see someone smoking a fag so you don't want to see someone smoking a vaporiser.

Justine's viewpoint highlights how space can be performative (Gregson and Rose 2000): because smoking has been excluded from certain spaces for so long, even the sight of something similar causes a visual offence. Others concurred, believing that the spatial rules for vaping should follow smoking: ‘you do it in your own space, that's what you do with cigarettes’ (Heather, smoker, ever‐vaped, 21). However, other participants disagreed referring to the more ephemeral qualities of vapour compared to cigarette smoke. Sarah was talking about visiting her parents’ home for the weekend and using her e‐cigarette indoors:

I've just started doing it and nobody said anything, they're like ‘no it's fine, it smells nice’ … and then I noticed it did create quite a mist at first but it just goes away, it's not like cigarette smoke where it just lingers and you can see this horrible grey mist, it just kinda goes away. Just a lot cleaner.

Dennis (2016) contends that it is the revulsion directed at second‐hand smoke that drives the norms of smokefree places. Sarah's account supports this as she notes that after emitting second‐hand vape, the air is not so adversely affected and quickly returns to a ‘clean’ state. Some participants expressed similar feelings and noted potentially de‐stigmatising effects of vaping instead of smoking, including avoiding yellowing teeth and fingernails and the smell of tobacco smoke on clothing. Others noted that second‐hand vape could also produce a more pleasant space, especially at home, compared to second‐hand smoke: ‘It's a better smell. Fair enough, it lingers, but I'd rather have a hazy room for a couple of minutes than it be yellow. Having to redecorate, having the whole house stinking’ (Steven, smoker, ex‐vaper, 20). This reflects the possibility for vaping to contribute towards a shedding of stigma (Coleman *et al*. 2016) from both participants’ bodies and homes. However, others responded negatively to second‐hand vape. Paul (smoker, vaper, 18), referring to the smells of a local vape shop, acknowledged the more pleasant odours, but still objected and drew on a place‐based smokefree imperative: ‘tell you what it smells nicer than cigarettes, but still no good, just toxic abomination … You shouldn't smell that in a shopping centre, you shouldn't smell anything in a shopping centre’.

The above discussion highlights the unresolved spatial rules and norms for vaping in the participants’ accounts. Two excerpts from the conversation between Ian, Patricia and Kelly demonstrated this how these views could quickly shift:

Kelly (never smoked, never vaped, 16) I don't actually know the rules on where you're allowed to use it or where you're not. I don't think anyone really does.

Ian (never smoked, ever‐vaped, 17) Yeah, I think because it's so new.

Kelly Like should we let them do that where there are children, should we not?

Patricia (ever‐smoked, never vaped, 19) Like in restaurants and stuff like that, I don't know.

Despite their uncertainty, they later continued to discuss reasons why people are upset at the use of e‐cigarettes. Despite all three being never vapers, a more resolved attitude towards vaping was settled on after Ian recounted a story of his mother defending her vaping after he had challenged her on using her e‐cigarette ‘everywhere’:

Ian You can't make opinions yet, it's just a thing.

Patricia Because if you're going to ask someone not to do it, then everybody would be like: ‘Why? There's nothing wrong with it, and you've got nothing to back up your opinion.’ It's just because you don't want them to do it.

Ian You don't like the notion of it, basically.

Encountering vaping was still generally new for these participants and the interactions which make‐up the performativity and the spatial rules (Dirksmeier and Helbrecht 2010), unlike those for smoking, were still developing. As participants mulled over these issues in the interviews a feeling of uncertainty was common, and viewpoints often changed during interviews as happened in the conversation above. However, participants almost always discussed vaping rules with reference to smoking rules demonstrating that: ‘performativity depends upon the sedimentation of the past’ (Ahmed 2004: 92).

## **Discussion and conclusion**

We found that participants knew little about the technical properties of e‐cigarettes and how they related to cigarettes. Like Roditis and Helpern‐Felsher (2015) who found that in a void of information about the relative harms of e‐cigarettes, young people may form positive opinions about vaping; our participants also turned to their embodied knowledge of smoking in order to develop vaping norms. However unlike Rodtiis and Helpern‐Felsher who found mostly positive reactions to e‐cigarettes, many of our participants’ internalised disapproval of smoking often extended to vaping due to the similar performativities in embodied and spatial practices. Therefore, some were already pre‐disposed to disapprove of vaping. Nonetheless, the performativity was still developing and had not yet become an embedded, shared set of social norms, a ‘stylised repetition of acts’ (Butler 2006: 191) and therefore some participants reacted more positively to vaping, as a practice that included similar performativities to smoking but without many of the negative or stigmatising aspects.

Vaping and its performativities therefore carried a number of potentialities for the participants and like recent findings from Rooke *et al*. (2016) there existed a range of opinions. Some noted the shedding of stigma (Coleman *et al*. 2016) that came with the different shapes and sizes of e‐cigarettes compared to cigarettes, supporting the devices as a potentially non‐stigmatising route to smoking cessation (Voigt 2015). Meanwhile the exhalation of a much less ‘polluting’ by‐product than second‐hand smoke (Dennis 2016), could provide an improved personal experience to smoking, possibly even ‘cool’ (Scheffels 2009) and furthermore improve the urban sensory landscape (Tan 2013). Conversely, similar hand‐to‐mouth performativities were viewed as negative by some who felt that it negated the cessation capabilities of e‐cigarettes. Likewise, the context of smoking denormalisation meant that – even if second‐hand vapour were less malodourous – some participants felt that all different smells were out of place in shared, public places. Spatial rules for vaping were therefore uncertain and so, while the geographies of smoking have ‘shrunk’ (Collins and Proctor 2011), the geographies of vaping are simultaneously shrinking and expanding.

While these qualitative data from a small purposive sample cannot be generalised to the Scottish or UK population, these findings may partly be explained by the participants being mostly from disadvantaged areas, and who reflected on the embeddedness and permissibility of smoking in their communities. Many participants preferred the familiarity of cigalike vaping rather than the more radically different performativities of vaping with a mod device or high‐end vapouriser. Furthermore, they noted the easy availability of cigalikes and the preclusive prices of more advanced models. Pricing and marketing may therefore be contributing to inequalities as only those with more disposable income are able to afford the devices perceived as most effective. Our findings are also limited to the specific geographical context and time when they were gathered. Given the rapidly evolving nature of e‐cigarette devices, this research highlights the need for continued qualitative research into how vaping is impacting upon smoking norms in different groups, especially with a focus on health inequalities.

The evidence in this paper suggests that the distinctions between vaping and smoking are unstable for the participants and so the normalisation pathways of the two behaviours are not wholly separate from one another. As Measham *et al*. (2016: 234) note, we are forced to interrogate our existing understanding of what it is that is being disapproved of in smoking and vaping, and ask: ‘to what extent does cultural acceptability or condemnation hinge on being able to see or smell consumption in public places?’. As Fairchild *et al* (2014: 295) point out, with reference to first generation ‘cigalikes’: ‘from the glowing tip to the smoke like vapour, e‐cigarettes seek to mimic the personal experience and public performance of smoking. But ironically, the attraction of the device is predicated on the continued stigmatization of tobacco cigarettes’. As devices initially designed to mimic smoking it would be remiss to assume that the connections between the two behaviours are not embroiled in the moral and ethical questions of denormalisation. Even with later generations that resemble conventional cigarettes less and less, performative similarities remain. From the perspective of those working in public health this ambiguity may help clarify what social and cultural barriers exist for smokers who have not turned to e‐cigarettes to help them quit, and contribute to developing more socially informed cessation interventions. Others however, may point to this research as evidence that smoking denormalisation messages have taken hold so much, that the young adults in this study may be wary of behaviours that involve the same performativity of cigarettes. From this perspective, endorsing e‐cigarettes could inadvertently lead to an implicit approval of all ‘smoking’ behaviours and a renormalisation of smoking. Regardless of interpretation, the performativity of vaping is far from settled as set of ingrained social practices. It will be particularly interesting to see where this performativity leads (or is led) and what the enduring consequences will be for smoking denormalisation.
